# Improving the *i*MM904 *S. cerevisiae *metabolic model using essentiality and synthetic lethality data

**DOI:** 10.1186/1752-0509-4-178

**Published:** 2010-12-29

**Authors:** Ali R Zomorrodi, Costas D Maranas

**Affiliations:** 1 Department of Chemical Engineering, The Pennsylvania State University, University Park, PA 16802, USA

## Abstract

**Background:**

*Saccharomyces cerevisiae *is the first eukaryotic organism for which a multi-compartment genome-scale metabolic model was constructed. Since then a sequence of improved metabolic reconstructions for yeast has been introduced. These metabolic models have been extensively used to elucidate the organizational principles of yeast metabolism and drive yeast strain engineering strategies for targeted overproductions. They have also served as a starting point and a benchmark for the reconstruction of genome-scale metabolic models for other eukaryotic organisms. In spite of the successive improvements in the details of the described metabolic processes, even the recent yeast model (i.e., *i*MM904) remains significantly less predictive than the latest *E. coli *model (i.e., *i*AF1260). This is manifested by its significantly lower specificity in predicting the outcome of grow/no grow experiments in comparison to the *E. coli *model.

**Results:**

In this paper we make use of the automated GrowMatch procedure for restoring consistency with single gene deletion experiments in yeast and extend the procedure to make use of synthetic lethality data using the genome-scale model *i*MM904 as a basis. We identified and vetted using literature sources 120 distinct model modifications including various regulatory constraints for minimal and YP media. The incorporation of the suggested modifications led to a substantial increase in the fraction of correctly predicted lethal knockouts (i.e., specificity) from 38.84% (87 out of 224) to 53.57% (120 out of 224) for the minimal medium and from 24.73% (45 out of 182) to 40.11% (73 out of 182) for the YP medium. Synthetic lethality predictions improved from 12.03% (16 out of 133) to 23.31% (31 out of 133) for the minimal medium and from 6.96% (8 out of 115) to 13.04% (15 out of 115) for the YP medium.

**Conclusions:**

Overall, this study provides a roadmap for the computationally driven correction of multi-compartment genome-scale metabolic models and demonstrates the value of synthetic lethals as curation agents.

## Background

*Saccharomyces cerevisiae *is the first eukaryote whose genome was fully sequenced and annotated [1]. It has since then been the focus of many genome-scale reconstruction efforts. Forster *et al *[2] reconstructed the first multi-compartment genome-scale metabolic model for yeast (i.e., *i*FF708). The model accounted for 708 open reading frames (ORFs) (~10.5% of all ORFs) and 1,175 metabolic reactions. The metabolic reactions for this model were compartmentalized between cytosol and mitochondria. Transport mechanisms between these two compartments as well as between the environment and cytosol were included in the model. Soon thereafter, the *i*ND750 model was introduced, which included five additional compartments (i.e., peroxisome, nucleus, golgi apparatus, vacuole and endoplasmic reticulum) by re-assessing the localization of gene products [3]. In a parallel study [4], the predictive capability of *i*FF708 was improved through a number of modifications in the biomass equation and the removal of blocked reactions (*i*LL672 model). Subsequently, another version of the yeast metabolic model with an improved description of the lipid metabolism containing 800 genes and 1,446 reactions (i.e., *i*IN800) was introduced by Nookaew *et al *[5]. These improvements to the yeast model culminated with the *i*MM904 model [6] that increased the size of *i*ND750 to 904 genes and 1,412 reactions. All these metabolic reconstruction efforts were carried out largely independently of one another using different data sources or in some cases different interpretations of the same literature evidence. This lack of consistency motivated the yeast systems biology community to consolidate all available metabolic models into a single consensus reconstruction and annotation model [7]. This model has been updated regularly since it was published and the latest version (i.e., Yeast 4.0) contains, in decompartmentalized form, 1102 reactions and 924 proteins [8].

Despite all these efforts there still exists a gap in the quality between the available metabolic reconstructions for yeast and corresponding models for microbial metabolism. Table 1 summarizes the quality metrics for the *E. coli *[9], *M. genitalium *[10], *B. subtilis *[11], *P. putida *[12], *H. pylori *[13] and *Salmonella *Typhimurium [14] latest genome-scale models when growth predictions are contrasted against experimental data for single gene deletions. As shown in this table, the accuracy (i.e., the fraction of correctly predicted lethal knockouts) of the *i*MM904 metabolic model of the yeast is significantly worse than any one of corresponding microbial models. This is partly caused by uncertainty in enzyme localization and inter-compartment metabolite transport in yeast [15]. This implies that a draft model reconstruction followed by even a detailed manual curation may not be a sufficient strategy to bring a eukaryotic genome-scale model to the same quality level as a microbial one. Here we explore the extent of model correction that can be brought about by systematically comparing the model predictions for single and multiple gene deletions with available experimental data.

**Table 1:** Accuracy of microbial metabolic models vs iMM904 model.

Microorganism	Name of the metabolic model	Specificity (%)	Reference
*Saccharomyces cerevisiae*	*i*MM904	38.8	[6] and this study
*Escherichiae coli*	*i*AF1260	73.4	[9]
*Mycoplasma genitalium*	*i*PS189	79.0	[10]
*Bacillus subtilis*	*i*BSU1103	89.3	[11]
*Pseudomonas putida*	-	74.5	[12]
*Helicobacter pylori*	*i*IT341 GSM/GPR	73.0	[13]
*Salmonella *Typhimurium	*i*MA945	66.7	[14]

Comparison of the fraction of correctly predicted lethal knockouts (i.e., specificities) of microbial genome-scale metabolic models against the yeast *i*MM904 model for single gene mutation experiments.

The established standard [15] for testing the accuracy of genome-scale metabolic models is to contrast the predicted growth phenotype of single mutant strains with the available experimental data under various growth conditions [6,9,16,17]. These comparisons result in four different outcomes [18]: GG or NGNG when both model and experimental data either imply growth (G) or no growth (NG) for the mutant strain, NGG when the model predicts that the gene deletion is lethal but the experiment shows that it is viable, and finally GNG when the model predicts that the mutant strain would be viable but *in vivo *observations show a lethal effect. Reed *et al *[19] proposed a systems analysis approach to restore growth for NGGs for a variety of growth media through the addition of appropriate metabolic and transport functions to the model. In another study, Satish Kumar *et al *[20] introduced GapFind and GapFill, to first identify metabolites that cannot be produced or consumed in the model under any uptake conditions and then bridge these gaps by adding missing functionalities to the model. Subsequently, another procedure termed GrowMatch [18] was proposed to reconcile both NGG and GNG growth prediction inconsistencies across different substrates (see Orth and Palsson [21] for a review). Notably, in the GrowMatch procedure [18], the GNG mismatches are corrected by modifying the metabolic model so as to convert them to NGNGs. Alternatively, Motter *et al *[22,23] suggested to reconcile GNGs by identifying candidate gene knockouts that can restore the growth of the mutants that were initially non-viable *in vivo*.

Recent studies have suggested that making use of not only single gene deletion information but also synthetic lethal pairs or (higher orders) can provide an additional layer for curation and validation of metabolic models. Harrison *et al *[24] showed that the investigation of falsely predicted synthetic lethals of *S. cerevisiae *can help to improve functional annotation. Furthermore, recent research by our group [25] demonstrated that mismatches between both growth and auxotrophy phenotypes of synthetic lethal predictions and *in vivo *observations can be used to provide 19 model correction hypotheses for the *i*AF1260 model of *E. coli*. In this paper, we modify and deploy automated approaches [18,20] for resolving falsely predicted single and multiple gene deletions under aerobic minimal and a complex (YP) medium, through the generation of appropriate model-correction hypotheses for the *i*MM904 model of the yeast. We chose *i*MM904 for this study because it contains biomass composition and reaction reversibility information making it suitable for performing FBA computations. Following the workflow presented in this paper we pinpointed 90 model corrections and identified 30 missing regulatory constraints with supporting literature evidence that almost doubled the prediction accuracy of the growth phenotype of single and double gene deletions under aerobic minimal and YP media for the *i*MM904 model. Examples of literature evidence include interaction of two proteins to support a modification in GPR associations, presence of a specific compound in the cell wall to corroborate its inclusion in the biomass equation, or gene expression data to confirm suppression of a gene under a given condition. The majority of modifications proposed here (i.e., 86% of them) remain relevant even for the latest update of the community yeast model (Yeast 4.0) [8].

## Results and discussion

### Single gene perturbations

**Figure 1:**
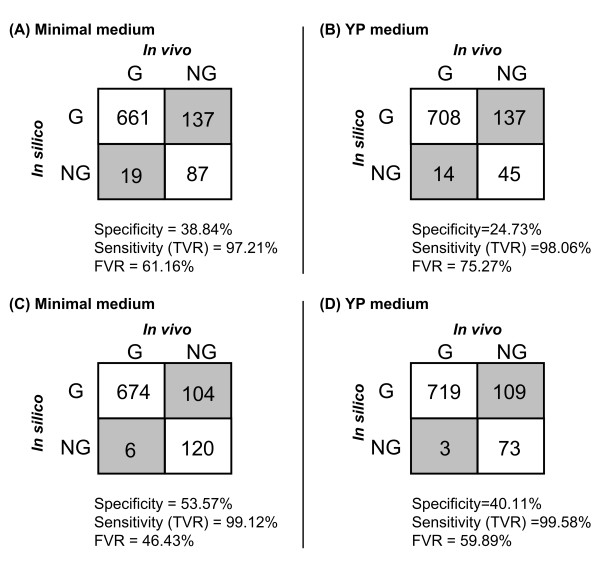
**Accuracy of the *i*MM904 model before and after modifications using single gene perturbations**. The number of false and correct predictions of the *i*MM904 model before (A and B) and after (C and D) applying the modifications for single gene mutations under minimal and YP media, respectively. Note that specificity = #NGNG/(#NGNG + #GNG), sensitivity or true viable rate (TVR) = #GG/(#GG + #NGG) and false viable rate (FVR) = #GNG/(#GNG + #NGNG).

Analysis of the impact of single gene or reaction deletions on the growth phenotype (for the *i*MM904 model) revealed 106 essential genes and 163 essential reactions for growth using a minimal medium as well as 57 essential genes and 92 essential reactions for the YP medium. We contrasted the predicted essential genes in both media to the experimental single gene deletion data reported by Kuepfer *et al *[4] to pinpoint any model inconsistencies. A summary of the model/experiment (mis)matches is given in Figure 1A and 1B (see Additional file 1 for details). We note that the two metrics specificity and sensitivity in this figure represent the fraction of correctly predicted lethal and viable mutants, respectively. Comparing the accuracy of the *i*MM904 model with *i*FF708 (specificity = 68.2%) [2,26] for growth using YP medium, we find that there is a significant reduction in specificity. This reduction is primarily due to uncertainties associated with the assignment of functionalities to compartments as well as the expansion of the model with less studied reactions, as pointed out earlier [15]. We applied the computational procedures described in the Methods section to reconcile these false predictions with the experimental growth. We note that all reaction and metabolite abbreviations throughout this manuscript are based on the *i*MM904 model.

#### Resolution of NGG inconsistencies

As denoted in Figure 1A and 1B, we identified 19 NGGs for the minimal and 14 for the YP medium. A summary of the pathways in which the NGGs for both growth media are involved is given in Additional file 1. The first step towards resolution of NGGs in the GrowMatch procedure is to look for alternate genes in yeast capable of carrying the same function but absent from the model or specific gene-protein-reaction (GPR) associations [18]. A bidirectional protein-protein BLAST (i.e., BLASTp) search against the *S. cerevisiae *genome revealed that seven of the genes involved in NGGs under the minimal medium and three under the YP medium share significant sequence similarity over the entire length of protein (i.e., forward and backward BLASTp expectation value of less than 10^-13^) with other ORFs in yeast. We however, rejected three such resolution strategies in the minimal medium and one in the YP medium due to contradicting experimental evidence (see Additional file 2 for details). Alternative correction strategies for the resolved NGGs were also explored. Two NGG inconsistencies under the minimal medium and one under the YP medium were resolved by relaxing the irreversibility of at least two reactions. For example, the deletion of gene *ADK1 *(YDR226W), which is involved in nucleoside salvage pathway, becomes non-lethal by treating any of the reactions ADPT (Adenine phosphoribosyltransferase), HXPRT (Hypoxanthine phosphoribosyltransferase (Hypoxanthine)), UPPRT (Uracil phosphoribosyltransferase) or GUAPRT (Guanine phosphoribosyltransferase) as reversible. The reversibility of these reactions was further supported based on the value of free Gibbs energy change, *ΔG*, or previous reports in literature [27-30] (also see Additional file 2). Notably, relaxing the irreversibility constraint on any of these reactions enables the production of prpp (5-phospho-alpha-D-ribose 1-diphosphate), which serves as a precursor for many biomass components.

Upon incorporation of these two global modifications in the model, we pursued the resolution of additional NGGs by adding transport reactions or reactions from the KEGG database [31] (see Methods). We were able to reconcile the growth inconsistencies for two NGGs in the minimal medium by this method. For example, *ADE8 *(YDR408C), which codes for reaction GARFTi (phosphoribosylglycinamide formyltransferas) was fixed by adding reaction R06974 (glycinamide ribonucleotide transformylase) to the model. GARFTi that serves as a step in the *de novo *purine nucleotide biosynthetic pathways is the only reaction in the model responsible for producing the essential metabolite fgam (N2-Formyl-N1-(5-phospho-D-ribosyl)glycinamide). Addition of reaction R06974 to the model provides an alternative way of producing fgam that can compensate for the absence of GARFTi. By performing the BLAST bi-directional test we identified an ORF in the yeast genome (i.e., *ADE2*) with very high sequence similarity (forward E-value = 3 × 10^-24 ^and backward E-value = 3 × 10^-24^) with the enzyme phosphoribosylglycinamide formyltransferase 2 (EC 2.1.2.-) catalyzing R06974 in *Methanococcus jannaschii*, which supports the presence of this reaction in yeast.

Except for two cases (i.e., *RIB1 *and *RIB4*), all other NGGs identified for the YP medium are also present and therefore corrected when considering the minimal medium. Both *RIB1 *(YBL033C) and *RIB4 *(YOL143C) are fixed not by modifying the *i*MM904 model but by adding missing compounds to the *in silico *YP medium description. Both of these genes are involved in riboflavin (vitamin B_2_) biosynthesis, which is a biomass precursor. Because yeast extract is reported to be a major source of vitamin B_2 _[32], we decided to add riboflavin to the list of components in the *in silico *YP medium. This renders the deletion of *RIB1 *and *RIB4 *non-lethal. Notably, the importance of correctly describing the medium composition in growth phenotype predictions has been raised in previous studies [26]. Overall, by using the GrowMatch procedure along with literature searches we were able to fix thirteen NGGs under the minimal medium and eleven under the YP medium (see Figure 1). The details of this analysis together with the evidence(s) found in support of each modification are provided as Additional file 2.

#### Resolution of GNG inconsistencies

As shown in Figure 1A and 1B, a total of 137 GNG inconsistencies in both minimal and YP media was identified with 128 of them jointly present. The distribution of these genes across different functional classes of metabolism revealed that most of them are involved in tRNA charging, oxidative phosphorylation and extracellular transport (see Additional file 1). Resolution of these GNGs by GrowMatch generally involves suppression of incorrectly present alternative production routes of biomass components in the model. The identified GNGs are divided into three major categories based on how they affect the flux distribution in the network [18]. The first category is comprised of genes coding for isozymes alluding that the deletion of these genes should not affect the reaction flux. A straightforward resolution strategy in this case is to suppress the other gene(s) whose product serves as isozyme for the coded reaction. An alternative hypothesis is to modify the corresponding GPR relationship so as to recast the deletion of that gene as lethal. These resolution strategies would be viable only if the coded reaction(s) are essential or form synthetic lethal(s) according to the model. For example, gene *ACO1 *(YLR304C), identified as a GNG under minimal medium, is an isozyme (with *ACO2*) for mitochondrial aconitase (ACONTm) and also independently catalyzes cytosolic aconitaase (ACONT) according to the *i*MM904 model. Notably, reactions, ACONT and ACONTm form a synthetic lethal pair under the minimal medium according to the *i*MM904 model. We did not find any evidence indicating that *ACO2 *is suppressed under aerobic glucose condition. Instead, we found that the protein coded for by *ACO2 *(Aco2p) is a putative mitochondrial aconitase isozyme, whose function has been assigned only based on the sequence similarity with Aco1p [33,34]. Therefore, we decided to remove *ACO2 *from the GPR for ACONTm thus rendering the deletion of *ACO1 *as lethal. This modification is consistent with the latest update of the community model [8]. Notably, another possibility is that *ACO2 *is correctly assigned to ACONTm, but *ACO1 *is involved in other unaccounted in the model essential functions (e.g., mitochondrial DNA maintenance [35]) in addition to its aconitase activity. We manage to fix five GNGs under the minimal medium and three under the YP medium by using this procedure.

The second category of GNGs contains genes that code for blocked reactions, i.e., the reactions that cannot carry any flux. This implies that even though the deletion of these genes will not affect the flux distribution *in silico*, their knockout is lethal *in vivo*. Resolution of these inconsistencies involves first unblocking the coded reactions and next rendering its deletion lethal under the examined conditions. Notably, 52 of such these GNGs code for reactions that are always blocked in the model mainly due to the presence of a no-consumption metabolite. One can thus resolve the inconsistency by exploring whether the no-consumption metabolite is a component of the biomass equation. Interestingly, we found that 21 of such these GNGs code for tRNA charging reactions that can be converted to NGNG consistencies by including the charged and uncharged tRNA molecules in place of the corresponding amino acids in the biomass equation. This strategy has been previously used in the reconstruction of the *Salmonella *metabolic model [14]. For example, the GNG prediction for *WRS1 *(YOL097C), which codes for the blocked reaction TRPTRS (Tryptophanyl tRNA synthetase), was corrected by including charged tRNA (trptrna) and uncharged tRNA (trantrp) as a reactant and product of the biomass reaction, respectively. This modification simultaneously unblocks reaction TRPTRS and renders it essential.

In many cases, including the no-consumption metabolite of a blocked reaction encoded by a GNG into the biomass equation was not a viable option as it also converted some GG consistencies to NGG mismatches (i.e., a conditional modification). Using the GapFind procedure [20] we found that such problem metabolites are all upstream no-consumption, implying that they can be fixed if their corresponding root (or one of its downstream) no-consumption metabolite(s) is resolved. Therefore, we tested whether these GNGs can be fixed globally, if the root or one of the downstream no-consumption metabolites is included as a component of the biomass equation. An additional seven GNGs corresponding to blocked reactions were fixed by this method but were not added to the list of proposed model modifications due a lack of experimental validation (see Additional file 2 for uncorroborated model changes). We note that the incomplete description of the biomass equation has been implicated as an important source of false model predictions in earlier studies [3,4,26]. Therefore, we exercised great caution when modifying the biomass equation to fix an inconsistency. Corrections were accepted only if solid corroborating literature evidence was found.

Two other GNGs were identified to correspond to a reaction that is blocked only under minimal conditions: Genes *MPH2 *(YDL247W) and *MPH3 *(YJR160C) encode isozymes catalyzing the transport of maltose from the extra-cellular environment to cytosol (reaction MALTt2 in *i*MM904). This reaction is blocked since no maltose is present in the minimal medium. Therefore, we explored whether these two genes are also involved in the transport of D-glucose (reaction GLCt1). A BLAST bi-directional analysis revealed that both of these genes have very high sequence similarity with glucose transport genes. Interestingly, we found that previous studies have reported on the involvement of *MPH2 *and *MPH3 *in mediating residual glucose uptake [36].

The third category of GNGs contains genes whose deletion affects the flux distribution in the network. To rectify these mismatches we employed the original GrowMatch procedure introduced in [18] and its modified version proposed in this study (see Methods) to identify the minimal number of genes/reactions whose suppression lower the biomass formation below the pre-specified viability threshold. We performed this analysis by allowing for up to two simultaneous gene/reaction suppressions for each GNG leading to the correction of 18 inconsistencies under the minimal and 19 under the YP medium. Twelve of these fixed GNGs in the minimal medium and fourteen in the YP were excluded from the list of corrected mismatches as we did not find any supporting evidence in the literature. Overall, we fixed 33 GNGs in the minimal and 28 in the YP medium by global modifications that do not conflict with any correctly predicted growth phenotype for single gene mutants. We note that 27 GNGs that appeared in both media were corrected by the same mechanisms (see the Additional file 2 for the exhaustive list of corrections).

Upon incorporating into the model only the corrections for NGGs and GNGs for which we found literature evidence, the accuracy of the *i*MM904 model was substantially improved. The number of correctly predicted lethal knockouts (i.e., specificity) out of a total of 224 was increased from 87 to 120 for the minimal medium and from 45 to 73 (out of a total of 182) for the YP medium. The corresponding false viability rate (FVR) was decreased from 61.16% to 46.43% for the minimal medium and from 75.27% to 59.89% for the YP medium. These results are summarized in Figure 1C and 1D.

### Double gene perturbations

**Figure 2:**
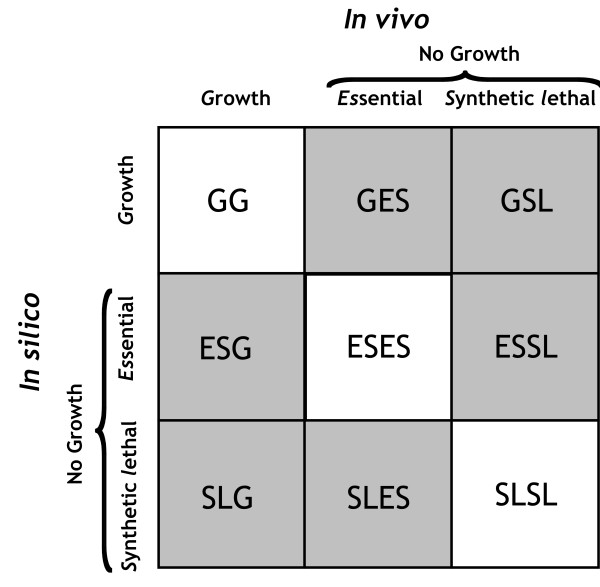
**Different types of mismatches between *in silico *predictions and *in vivo *observations for double gene perturbations**. The abbreviations G, ES and SL in this figure refer to *Growth*, *Essential *and *Synthetic Lethal*, respectively. Here, 'No Growth' can be due to either essentiality or synthetic lethality of single or double gene deletions.

We employed the SL-Finder procedure [25] to identify the set of all synthetic lethal (SL) gene pairs under both minimal and YP media based on *i*MM9904 model of the yeast. This analysis led to identification of 97 SL pairs in the minimal medium and 42 in the YP medium. Model SL prediction inconsistencies were identified by contrasting against the available experimentally identified SLs (see Additional file 1 for a complete list of predicted and experimentally identified synthetic lethal interactions). As shown in Figure 2, this comparison reveals a number of additional ways that model and experiment may differ in their predictions. Notably, the "no growth" phenotype in this case could be due to either essentiality (ES) or synthetic lethality (SL) of the gene deletions. For example, GES and GSL inconsistencies refer to cases where the *in silico *deletion of a gene pair is not lethal (i.e., *G*rowth) but *in vivo *they are lethal due to gene essentiality or synthetic lethality (i.e., *ES*sential or *S*ynthetic *L*ethal). Similarly, ESG and ESSL represent mismatches where the single deletion of one of the genes *in silico *is lethal (i.e., *ES*sential), however, their simultaneous deletion *in vivo *results in either a viable strain (i.e., *G*rowth) or a lethal phenotype (i.e., *S*ynthetic *L*ethal), respectively. Finally, SLG and SLES denote inconsistencies where the model implies that only the double gene mutation is lethal (i.e., *S*ynthetic *L*ethal) but experimental observations support either growth (G) or lethality of any of the two single gene deletions (i.e., *ES*sential), respectively.

In the following we describe how the restoration of each type of these inconsistencies can be used to improve the predictive capability of the *i*MM904 model. In analogy to the definition of specificity for single gene mutations, we define the specificity for double gene perturbations as the fraction of correctly predicted synthetic lethals (SLSL), i.e., S*pecificity = #SLSL/(#SLSL + #ESSL +# GSL)*. We do not comment on the resolution of GES and ESG mismatches, as they are completely equivalent to GNG and NGG inconsistencies. In addition, we note that all the model corrections are based on the currently available incomplete list of experimentally identified SLs.

#### Resolution of GSL inconsistencies

The GSL inconsistencies denote cases where the model implies growth under the simultaneous deletion of two genes while experimental results show a lethal effect. A total of 104 GSLs in the minimal medium and 98 in the YP medium were identified. We note that GSLs for double perturbations can be treated similarly to the GNG mismatches for single perturbations with the only difference that "no growth" in GNGs is due to the essentiality of single genes whereas in GSLs it is the result of synthetic lethality.

The strategy for fixing GSLs involved in coding for isozymes is to repress the other genes coding for that isozyme under the examined condition or modify the GPR associations. When both genes code for isozymes for the same reaction this resolution strategy is viable only if that reaction is essential *in silico*. Alternatively, when two genes are associated with two (or more) different reactions they must form a synthetic lethal. We note that the gene repressions for this group of GSLs can be inferred either manually by inspection of the GPR associations for each inconsistency (as we did for GNGs), or automatically, by using the modified version of the GrowMatch (see Methods). As an example, the modified GrowMatch procedure suggests that the GSL (*ASN1*,*ASN2*) can be fixed by suppressing gene YML096W. By reviewing the GPR relationships we found that genes *ASN1 *(YPR145W) and *ASN2 *(YGR124W) together with YML096W provide isozymes for catalyzing the essential reaction ASNS1 (asparagine synthase (glutamine-hydrolysing)). Therefore, suppressing gene YML096W will render the simultaneous deletion of *ASN1 *and *ASN2 *lethal. Note, however, that we did not find any evidence in support of YML096W suppression under aerobic minimal conditions, but we found that this gene is coding for a putative protein of unknown function and has been assigned to ASN1 only based on the sequence similarity [37-39] prompting us to remove YML096W from the GPR association to ASN1. This example demonstrates that the identified gene suppressions by GrowMatch may allude to incorrect assignment of genes to reactions.

Resolution of GSL inconsistencies coding for the blocked reactions is more complicated than for GNGs. Different cases need to be examined: (1) If both genes appear in the GPR relationship for the same blocked reaction then we revert to the method discussed for fixing GNGs. (2) If the two genes are coding for two (or more) different reactions that are all blocked because of the same root no-consumption metabolite then exploring the addition of the (common) downstream, or the corresponding root problem metabolite, to the biomass equation as a reactant may fix the inconsistency. (3) If the two genes are coding for reactions that are blocked because of different root no-consumption metabolites or one of the genes codes for a non-blocked reaction then we cannot reconcile the growth inconsistency by simply modifying the biomass reaction. To resolve such GSLs, the GapFill procedure is applied first to correct the root no-consumption metabolites by adding a consumption or export pathway to the model, thereby unblocking the coded reactions. With this modification, these GSLs can now be treated as those where deletion of each single gene will change the flux distribution *in silico*. We could only fix one GSL inconsistency by modifying the biomass equation: *KRE6 *(YPR159W) and *SKN1 *(YGR143W) that provide isozymes for the blocked reaction 16GS (1,6 β-glucan synthase) were fixed by including 16BDglcn (1,6 β-D-glucan) a root no-consumption metabolite in the biomass equation as a reactant. This modification is corroborated by the previous reports showing that 1,6 β-D-glucan is a key component of the yeast cell wall [40-42].

**Figure 3:**
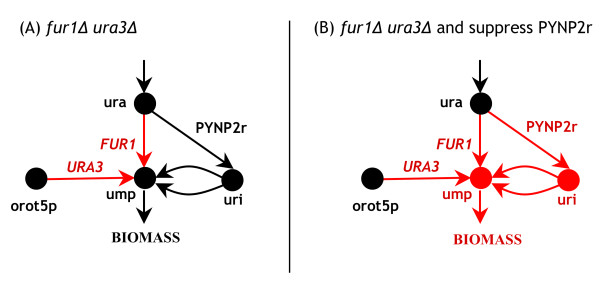
**Resolution of a GSL through reaction suppressions**. (A) *FUR1 *and *URA3 *form a GSL since based on the *i*MM904 model there exists alternative pathways for producing the biomass precursor ump in the absence of these genes. (B) By suppressing (or removing) the reaction PYNP2r, all the production routes towards the biomass precursor ump is blocked, rendering the simultaneous deletion of *FUR1 *and *URA3 *lethal.

Reconciling GSLs where disruption of each single gene affects the *in silico *flux distribution, involves identifying missing regulatory constraints on genes/reactions by using the GrowMatch procedure and its modified version. We followed this analysis by allowing for up to two simultaneous gene/reaction suppressions for each GSL and considered only the global modifications. For example, genes *ZWF1 *(YNL241C) and *RPE1 *(YJL121C) are both involved in the pentose-phosphate pathway and form a GSL (under minimal medium). The deletion of either *ZWF1 *or *RPE1 *will change the flux distribution *in silico*. Application of the GrowMatch to fix this inconsistency suggests suppressing reaction G6PDH2er (glucose-6-phosphate dehydrogenase [endoplasmic reticulum]) which is identical to the reaction catalyzed by Zwf1p in the cytosol (i.e., G6PDH2). This suggests that glucose-6-phosphate dehydrogenase activity in the endoplasmic reticulum is not sufficient to compensate for its deficiency in cytosol in an *RPE1 *mutant background. Interestingly, it has been shown [43] that mammalian cells have two sets of enzymes catalyzing the reactions of the pentose phosphate pathway (including glucose-6-phosphate dehydrogenase) with the more active set in the cytoplasm and the less active in the endoplasmic reticulum. If this holds true for yeast then this could resolve the inconsistency. An alternative hypothesis for fixing the inconsistency is that *ZWF1 *independently codes for glucose-6-phosphate dehydrogenase activity in the endoplasmic reticulum (i.e., reaction G6PDH2er) as well as in the cytosol (i.e., reaction G6PDH2). In another example, *FUR1 *(YHR128W) and *URA3 *(YEL021W) form a GSL. They catalyze two reactions that serve as alternative production routes for ump (uridine mono-phosphate) a biomass precursor. Application of the GrowMatch procedure to fix this GSL suggests repressing reaction PYNP2r (pyrimidine-nucleoside phosphorylase (uracil)). According to the *i*MM904 model, reaction PYNP2r provides a source of uri (uridine), which subsequently is converted into ump. Suppression of PYNP2r will thus render the simultaneous deletion of *FUR1 *and *URA3 *lethal by blocking all production routes for the biomass precursor ump (see Figure 3). Note that since PYNP2r does not have any gene association in the model, its suppression to fix the GSL inconsistency also raises the possibility that this reaction is erroneously included in the model and should thus be removed.

Not surprisingly all the identified suppressions for correcting GSLs for the minimal medium were also valid for the YP medium. We identified only one case of a gene suppression to resolve a GSL, which was valid for only the YP medium. Genes *MET12 *(YPL023C) and *MET13 *(YGL125W), identified as a GSL in both minimal and YP media provide isozymes for reaction MTHFR3 (5,10-methylenetetrahydrofolatereductase (NADPH)), which is involved in folate metabolism. Application of the modified GrowMatch to resolve this inconsistency suggests suppression of *CYS3 *(YAL012W) in both minimal and YP media. However, suppressing *CYS3 *under the minimal medium will change some GGs to NGGs (i.e., it is a conditional modification) whereas, it is a global suppression in the YP medium. Interestingly, in support of *CYS3 *suppression in the YP medium, it was reported before that this gene is cystein, methionine and glutathione suppressed [44].

**Figure 4:**
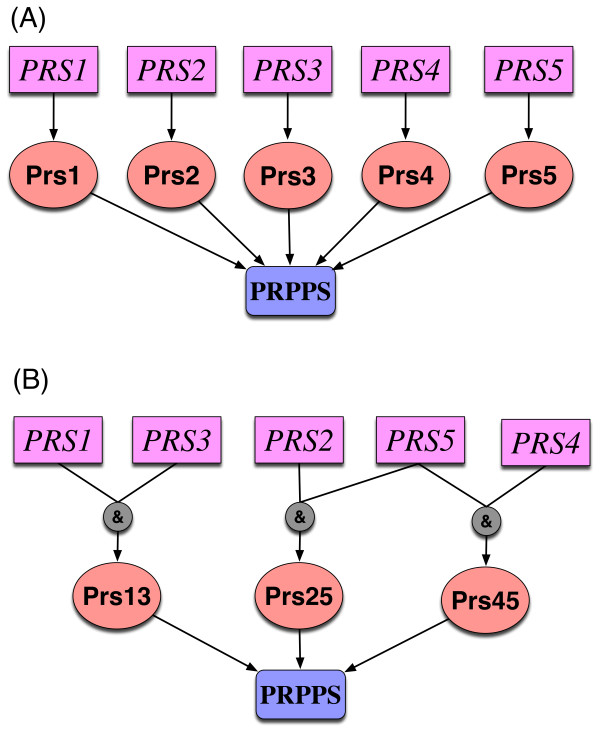
**Change of GPR for reaction PRPPS**. The GSL inconsistencies (*PRS1*,*PRS5*), (*PRS3*,*PRS5*), (*PRS1*,*PRS2*,*PRS4*) and (*PRS3*,*PRS2*,*PRS4*) imply that the GPR for reaction PRPPS should be changed from (*PRS1 *OR *PRS2 *OR *PRS3 *OR *PRS4 *OR *PRS5*) to (*PRS1 *AND *PRS3*) OR [(*PRS2 *OR *PRS4*) AND *PRS5*)].

Finally, in some cases GSL mismatches allude to incorrect GPR relationships in the model. For example, we found four GSLs (under minimal medium) involved in a GPR that implicates genes *PRS1 *through *5*. The first two include two genes (i.e., (*PRS1*,*PRS5*) and (*PRS3*,*PRS5*)) and the other two contain three genes (i.e., (*PRS1*,*PRS2*,*PRS4*) and (*PRS3*,*PRS2*,*PRS4*)). All these genes provide isozymes for the essential reaction PRPPS (phosphoribosylpyrophosphate synthetase) according to the *i*MM904 model. The inconsistency between model predictions and experimental observations can be resolved by changing the GPR relationship from (*PRS1 *OR *PRS2 *OR *PRS3 *OR *PRS4 *OR *PRS5*) to (*PRS1 *AND *PRS3*) OR [(*PRS2 *OR *PRS4*) AND *PRS5*)] to render the simultaneous deletion of each of the aforementioned gene pairs/triple combinations lethal (see Figure 4). Interestingly, in a previous study a strong interaction between *PRS1 *and *PRS3 *as well as between *PRS5 *and either *PRS2 *or *PRS4 *has been described [45]. In total, we fixed eleven GSLs in the minimal medium and five in the YP medium by converting them to SLSL consistencies by relying on literature vetted global modifications (see Additional file 2 for details).

#### Resolution of ESSL inconsistencies

The ESSL inconsistencies refer to cases where the model predicts that one or both of the genes are essential even though the experimental results show that they form a SL. Based on the available data in literature, we identified 13 ESSLs containing seven *in silico *essential genes in the minimal medium and nine ESSLs containing five *in silico *essential genes in the YP medium (see Additional file 1). Not surprisingly, all these *in silico *essential genes were part of the previously identified NGG mismatches for the single gene mutations. Consequently, a direct resolution strategy to fix the ESSLs is resolving the NGGs for the single gene perturbations. Following this route, we found that three ESSLs in the minimal medium and one in the YP medium were automatically rectified and converted to SLSLs through the resolution of NGGs (see Additional file 2 for details). However, the rest of them were not resolved, instead they were converted into new GSL mismatches. Except for one case, application of the methods discussed in the previous section for resolving GSL mismatches did not identify any global or literature supported correction strategies.

An interesting example of ESSL mismatches is the gene pair *CHO2 *(YGR157W) and *OPI3 *(YJR073C) where each gene is essential *in vivo *[4]. *CHO2 *catalyzes the first step in conversion of pe_SC (phosphatidylethanolamine) to pc_SC (phosphatidylcholine) in phospholipid biosynthesis pathway (reaction PETOHM_SC in the *i*MM904 model), whereas, *OPI3 *catalyzes the last two steps (reactions MFAPS_SC and PMETM_SC). We were not able to fix neither *CHO2 *nor *OPI3 *as separate NGGs by any of the methods discussed previously. However, the fact that these two genes form a SL *in vivo *suggests that they may act as isozymes for reactions PETOHM_SC, MFAPS_SC or PMETM_SC. Interestingly, by mining the literature we found that previous studies have already demonstrated that *OPI3 *can partially contribute as an isozyme in catalyzing reaction PETOHM_SC [46] implying that the GPR for this reaction should be changed to (*CHO2 *OR *OPI3*). There were no reports implicating *CHO2 *as an isozyme for catalyzing reactions MFAPS_SC or PMETM_SC. However, previous studies have reported that *S. cerevisiae *is flexible with respect to phospholipid composition and can substitute pe_SC, ptdmeeta_SC (phosphatidyl-monomethylethanolamine) or ptd2meeta_SC (phosphatidyl-dimethylethanolamine) for pc_SC to a substantial extent [46-49]. In order to capture this lack of specificity we removed pc_SC from the biomass reaction and instead added a proxy *phospholipid *compound with the same stoichiometric coefficient as that for pc_SC. Subsequently, we added four hypothetical reactions to the model that produce the phospholipid from any of pc_SC, pe_SC, ptdmeeta_SC or ptd2meeta_SC. Note that these modifications to the model were all global and fixed the inconsistencies for *OPI3 *as a NGG as well as (*CHO2*, *OPI3*) as an ESSL mismatch.

#### Resolution of SLG inconsistencies

The SLG inconsistencies represent a mismatch where the two genes form an *in silico *SL, however, their simultaneous deletion results in a viable strain. SLG mismatches for double gene perturbations can be viewed as NGG mismatches for single gene perturbations in the sense that they both imply that certain functionalities are missing in the model. The only difference between NGGs and SLGs is that the "no-growth" in NGGs is due to the essentiality of single genes whereas in SLGs it is the result of synthetic lethality of gene pairs. Therefore, we simply adapt the same procedure that we used for fixing NGGs to resolve SLG inconsistencies.

We found and resolved one case of such an inconsistency in both minimal and YP media. This SLG pertains to genes *PGM1 *(YKL127W) and *PGM2 *(YMR105C) that code for isozymes of reaction PGMT (phosphoglucomutase) involved in glycolysis/gluconeogenesis. The simultaneous deletion of *PGM1 *and *PGM2 *is lethal based on the *i*MM904 model since glygogen, a biomass precursor, cannot be produced in the absence of these two genes. Application of the GrowMatch procedure to fix this SLG, did not lead to any literature-supported correction strategy. We next mined the literature for a possible isozyme for this reaction and identified that gene *PGM3 *(YMR278W) is known to catalyze the interconversion of glucose-1-phophate to glucose-6-phosphate [50], however, it is missing in the *i*MM904 model. The addition of *PGM3 *to the GPR for reaction PGMT (i.e., *PGM1 *OR *PGM2 *OR *PGM3*) renders the simultaneous deletion of *PGM1 *and *PGM2 *non-lethal.

#### Resolution of SLES inconsistencies

The SLES mismatches denote cases where the model predicts that only the simultaneous deletion of both genes is lethal whereas one of the genes (or both) is essential *in vivo*. This implies that deletion of one of these two genes cannot be compensated for by the other gene under the experimental conditions. Therefore, SLES inconsistencies are rectified by suppressing in the model the gene that is not essential *in vivo*. Essential genes participating in *in silico *SLs yield GNG inconsistencies. Therefore, the resolution of these GNGs also fixes the SLES mismatches for the double gene mutations. We identified 59 SLES mismatches for the minimal medium and 42 for the YP medium. Fifty one of these SLESs in the minimal and two in the YP medium were fixed by using the global modifications found for GNGs. For example, gene *IPP1 *(YBR011C) which codes for PPA (inorganic diphosphatase involved in oxidative phosphorylation) forms a SL pair under both minimal and YP medium with gene *IPP2 *(YMR267W) that codes for the same reaction in mitochondria (reaction PPAm). However, *IPP1 *has been found to be essential *in vivo *[4]. Therefore, to resolve this inconsistency, we conditionally suppress gene *IPP2 *under aerobic conditions. Interestingly, by investigating the available expression data for these genes we found that the expression level of *IPP1 *under aerobic conditions with glucose as the carbon source is almost 37 times higher than that for *IPP2 *[51], which may explain why *IPP2 *is not able to compensate for the deletion of *IPP1*.

In another example, gene *PGK1 *(YCR012W), which is associated with reaction PGK (phosphoglycerate kinase) involved in glycolysis/gluconeogenesis, participates in as many as 39 *in silico *SLs under minimal medium. However, it has been found to be essential *in vivo *[4]. This implies that suppressing at least one of the 39 genes forming a synthetic lethal with *PGK1 *would resolve all these SLESs. Although, we did not find any evidence confirming this resolution hypothesis for any of these genes except for *PCK1 *(YKR097W), which is known to be suppressed in presence of glucose [52,53]: this gene is involved in gluconeogenesis, a process allowing yeast to synthesize glucose from non-carbohydrate precursors such as ethanol or glycerol. Notably, suppression of *PCK1 *in the *i*MM904 model will block production of three biomass precursors, i.e., phe-L (L-Phenylalanine), trp-L (L-Tryptophan) and tyr-L (L-Tyrosine) in the absence of *PGK1*.

Overall, the resolution of model inconsistencies for double gene deletions improved the specificity of *i*MM904 model from 12.03% to 23.31% (out of a total of 133) for the minimal medium and from 6.96% to 13.04% (out of a total of 115) for the YP medium. It is worth noting that these corrections are based on only the incomplete list of SL data available in literature.

### Auxotrophy inconsistencies

These mismatches refer to cases where the essentiality of single gene deletions or synthetic lethality of double gene knockouts are in agreements with *in vivo *observations, however, the model predictions for supplementation rescue (i.e., auxotrophy) scenarios are inconsistent with experimental data. We found seven such these inconsistencies under minimal and five under YP medium, respectively, for correctly predicted essential genes, as well as four under both minimal and YP media for SLSL predictions (see Additional file 1 for a complete list). Notably, for all of these mismatches, the experimental results show that the single or double gene mutant strains can restore growth if additional compounds are added to the growth medium, while the model predictions imply that these genes remain essential or synthetic lethal even in the presence of these compounds. These inconsistencies can be treated in exactly the same way as the NGG or SLG mismatches were treated, since they refer to the functionalities that are missing in the model but present under experimental conditions.

As an example, it has been reported that a strain containing the *FOL1 *(YNL256W) deletion can grow if the medium is supplemented with folic acid [54]. Nonetheless, folic acid is not included in the list of metabolites in *i*MM904 model. By adding folic acid as well as exchange and transport reactions (between cytosol and extracellular environment) to the *i*MM904 model *FOL1 *remains essential even though folic acid is allowed to be taken up. GrowMatch suggested addition of any of the two reactions R00937 (5,6,7,8-tetrahydrofolate:NAD+ oxidoreductase) or R02236 (dihydrofolate:NADP+ oxidoreductase) from the KEGG database to the model so as to connect folic acid to rest of the network. Interestingly, by searching the KEGG database we found that the enzyme catalyzing these two reactions is present in yeast and the gene coding for this enzyme (*DFR1*: YOR236W) is already present in the *i*MM904 model.

The gene pair *HMG1 *(YML075C) and *HMG2 *(YLR450W), which forms a SLSL under both minimal and YP media. is an example of auxotrophy mismatches for double gene perturbations: although *in vivo *observations show that a mutant strain lacking these two genes can be rescued through addition of mev-R (mevalonate) to the growth medium [55], *in silico *predictions imply that their double deletion is still lethal even in the presence of mev-R. The addition of an exchange and transport reaction between cytosol and extracellular environment for mev-R to the model resolves this auxotrophic inconsistency. Notably, the addition of this import pathway for mev-R to the model also fixed the auxotrophy inconsistency for the essential gene *ERG10 *(YPL028W), which is involved in mevalonate biosynthesis.

**Figure 5:**
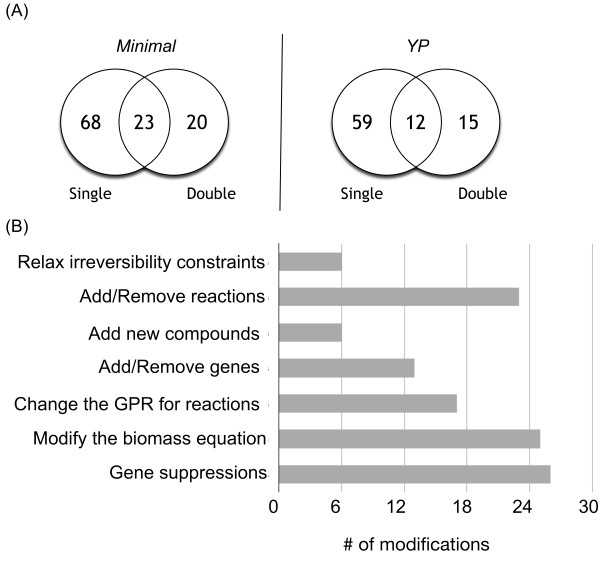
**A summary of all modifications to the *i*MM904 model using the single and double gene perturbations**. (A) Venn diagrams for minimal and YP media representing the number of modifications involved in fixing the inconsistencies associated with single or double gene deletions or both. (B) A Bar chart demonstrating the number of each type of modifications made to the *i*MM904 model.

Overall, upon including only the global modifications for which a supporting evidence was found, we could fix three auxotrophy inconsistencies for essential genes as well as one for SLs under both minimal and YP media (see Additional file 2 for details). A summary of all the suggested modifications for the *i*MM904 model by using all types of inconsistencies for single and double gene perturbations is given in Figure 5. The revised *i*MM904 model is also available in the Systems Biology Markup Language (SBML) as Additional file 3.

## Conclusions

We identified 120 corrections with supporting evidence to the *i*MM904 metabolic model of yeast by using essentiality and synthetic lethality data. Previous studies geared towards improving the predictive ability of metabolic models have used growth phenotype inconsistencies for single gene mutation experiments in microbial systems such as *E. coli *[18,19]. Here, we go a step further by demonstrating the utility of synthetic lethality data for improving the accuracy of a multi-compartment metabolic model for a eukaryotic organism. This revealed missing or erroneously present metabolic functions in the model that could not be captured by only single gene perturbations. In addition, we found that in some cases fixing a mismatch for double gene deletions automatically fixes one or more mismatch(s) for single gene perturbations. This was illustrated for the NGG, *CHO2 *for which none of the mechanisms proposed to resolve the NGGs were found to be successful. Approximately, 20% of the total suggested corrections for the *i*MM904 model use information from both single and double gene perturbations whereas 17% of them were exclusively discerned from double gene perturbations. A far larger contribution of synthetic lethals in providing model refinement strategies is thus expected as more synthetic lethality data are becoming available.

The high number of GNG and GSL inconsistencies identified in this study runs contrary to the general perception that predictive inaccuracy of genome-scale metabolic models is primarily due to missing metabolic capabilities. It appears that the presence of not properly restricted to specific conditions functionalities in the model is the largest contributor to inconsistent predictions. Application of GrowMatch to eliminate and/or properly regulate these functionalities led to the identification of 30 growth medium-specific regulatory constraints. These growth prediction inconsistency-based constraints complement existing regulatory constraints based on gene expression data [44,56,57].

In this study we considered not only essentiality and synthetic lethality predictions but also disagreements in auxotrophy complementation. We also demonstrated that the identified growth-phenotype discrepancies are sometimes due to an incorrect or incomplete *in silico *description of the complex growth medium not the inaccuracy of the metabolic model. Overall, we significantly improved the predictive capability (i.e., specificity, sensitivity and false viability rate) of the *i*MM904 model for essentials and synthetic lethals by incorporating a minimum of 112 (out of 120) suggested corrections. All of these modifications are global as they do not invalidate any of the correct model predictions. The proposed corrections span a wide array of changes to the model including relaxation of the irreversibility constraints on existing reactions in the model, adding new reactions, compounds or genes to the model, modifying the biomass equation, changing the GPR associations and medium-specific regulatory constraints. As we found independent corroborating evidence for the proposed corrections, the vast majority (i.e., 103) of them remain relevant even for the latest update of the community yeast model (Yeast 4.0) [8]. This includes 73 model refinement strategies as well as 30 identified medium-specific regulatory constraints. Of the remaining modifications to the *i*MM904 model, twelve were also independently incorporated in Yeast 4.0 whereas five are different from the ones adopted in Yeast 4.0. A comparison of the suggested corrections with Yeast 4.0 is given in Additional file 2.

In addition to model refinement strategies with supporting evidence, we identified more than 60 other global modifications for which there was neither conflicting nor supporting evidence (see Additional file 2 for a complete list). These modifications can be treated as testable hypotheses for which experiments can be designed to prove/disprove their validity. Overall, our study demonstrates the value of bringing to bear multi-gene deletion data to further improve the predictive capability of genome-scale metabolic models. The availability of high-throughput experimental techniques [58-61] as well as efficient computational tools [25,62,63] to elucidate synthetic lethal interactions opens the door to rapidly reveal additional model deficiencies. The model refinement approaches presented in this study are versatile enough to be employed for a wider range of experimental conditions (e.g., other growth media) or synthetic lethal interactions of increasing size (e.g., triples, quadruples, etc).

## Methods

We applied the SL Finder procedure developed by Suthers *et al *[25] to the *i*MM904 model of *S. cerevisiae *[6] comprised of 904 genes and 1,412 reactions to determine the set of essential genes/reactions as well as different orders of synthetic lethals. All simulations were performed for aerobic growth on two different media with glucose as the sole carbon source: minimal medium and a complex (yeast-extract peptone or YP) medium. The *in silico *minimal medium contains ammonium, sulfate and phosphate as nitrogen, sulfur and phosphor sources, respectively, as well as necessary salts (such as Na and K). In addition to all these components the *in silico *YP medium contains all 20 amino acids and all four nucleotides [3]. All simulations were performed for a strain auxotrophic for methionine, leucine, histidine and uracil to closely mimic the conditions used in experimental studies [4]. This auxotrophy was simulated by deleting genes (*his3Δ leu2Δ met15Δ ura3Δ*) and supplementation of the growth medium with the missing nutrients at non-limiting but low levels [6]. In addition, trace amounts of other essential compounds that are present in the experimental growth medium including 4-aminobenzoate, biotin, inositol, nicotinate, panthothenate and thiamin were added to the *in silico *media [6]. Consistent with Suthers *et al *[25] we chose 1% of the maximum theoretical biomass yield as the viability threshold for computational identification of lethal knockouts. The upperbound for all reactions was set to 1000, whereas, the lowerbound was set to 0 for irreversible reactions and -1000 for reversible reactions. The maximum rate of the glucose uptake was set to 10 mmol gDW^-1^h^-1 ^and the aerobic condition was modeled by limiting the oxygen uptake rate to 2 mmol gDW^-1^h^-1 ^[6]. The uptake rate for all other source metabolites was also set to 1000 mmol gDW^-1^h^-1^. The flux of non-growth associated ATP maintenance was fixed at 1 mmol gDW^-1^h^-1 ^[6]. We employed the GrowMatch procedure [18] to reconcile the growth phenotype discrepancies for the NGGs, and modified it to fix GNGs (in addition to using its original form). All these algorithms were adapted for resolving the inconsistencies associated with double gene perturbations. In the following we provide a brief overview of GrowMatch and describe in detail its modified version.

### The Modified GrowMatch procedure

The GrowMatch procedure relies on two separate procedures to resolve NGG and GNG growth prediction inconsistencies. The NGGs are fixed one-by-one by minimally perturbing the original metabolic model using three mechanisms including (*i*) relaxation of the irreversibility constraints on reactions in the model, (*ii*) addition of new reactions from external databases such as KEGG [31] to the model and (*iii*) allowing for direct import/export of metabolites into/out of the cell, and for multi-compartment models [18], addition of transport reactions between compartments and cytosol. Alternatively, the GNG mismatches are corrected by identifying the minimal set of suppression constraints for reactions or transport mechanisms that lower the maximum biomass yield of the network below a pre-specified viability threshold. The suggested modifications by GrowMatch are referred to as *global *if they do not conflict with any correct model predictions, and they are called *conditional *otherwise.

Here, we modified the GrowMatch procedure to identify the minimal number of suppressed genes, rather than reactions, that negate the biomass formation below the viability threshold. This can be done by defining two sets of binary variables one for genes and one for reactions and then relating these two binary variables in such a way that it reflects the specific GPR associations [25]. For the reactions with no gene associations a fictitious gene coding for that reaction can be assumed. An alternative way to avoid introducing a new set of binary variables, and as a result reduce the computational burden further, is to directly correlate the binary variables for genes to the reaction fluxes. To this end, we first, define the following sets:

$$\begin{array}{l} I=\{i|i=1,2,...,N\}=\text{set of metabolites}\\ J=\{j|j=1,2,...,M\}=\text{set of reactions }\\ K=\{k|k=1,2,...,G\}=\text{set of genes} \end{array}$$

where, *N*, *M *and *G *denote the total number of metabolites, reactions and genes in the network, respectively. To simulate the gene knockouts, a binary variable *w_k_*, representing if a gene *k *should be deleted is defined as following:

(1) $$w_{k}=\left\{ \begin{array}{l} 0,\text{ if gene }k\text{ is deleted}\\ \text{1, if gene }k\text{ is active} \end{array}\right.\;\forall \;k\in K$$

The impact of gene knockouts on reactions through the GPR relationships can be mathematically described by using appropriate constraints relating *w_k _*to the reaction fluxes, *v_j_*. Let *LB_j _*and *UB_j _*represent the lowerbound and upperbound on a reaction *j*, respectively. Different cases for the GPR relationships can then be considered:

(*i*) A single gene *k *codes for the enzyme catalyzing a reaction *j*. This can be easily incorporated into our mathematical framework by using the following constraint:

(2) $$w_{k}.LB_{j}\leq v_{j}\leq w_{k}.UB_{j}$$

(*ii*) Two genes *k_1 _*and *k_2 _*form a single multi-protein enzyme to enable a reaction *j*. This case, which is recast as a logic AND relationship between the genes *k_1 _*and *k_2_*, can be enforced by the following set of constraints:

(3) $$\left\{ \begin{array}{l} w_{k_{1}}.LB_{j}\leq v_{j}\leq w_{k_{1}}.UB_{j}\\ w_{k_{2}}.LB_{j}\leq v_{j}\leq w_{k_{2}}.UB_{j} \end{array}\right.$$

Note that based on these equations, if at least one of $w_{k_{1}}$ or $w_{k_{2}}$ is zero, then the flux through reaction *j *is forced to zero. This set of constraints can be easily generalized for multi-protein enzymes with more than two genes involved.

(*iii*) The genes *k_1 _*and *k_2 _*provide isozymes catalyzing a reaction *j*. This case, indicates a logic OR relationship between the genes *k_1 _*and *k_2 _*to enable the reaction *j*, and can be mathematically expressed by the following constraints:

(4) $$\left\{ \begin{array}{l} (w_{k_{1}}+w_{k_{2}}).LB_{j}\leq v_{j}\leq (w_{k_{1}}+w_{k_{2}}).UB_{j}\\ LB_{j}\leq v_{j}\leq .UB_{j} \end{array}\right.$$

Note that according to these equations, *v_j _*is forced to zero only if both genes are knocked out, i.e., $w_{k_{1}}$ = $w_{k_{2}}$ = 0. For the case, where both genes are present ($w_{k_{1}}$ = $w_{k_{2}}$ = 1), the second constraint is more binding and will restrict *v_j _*to fall within its defined lower and upperbound. This approach can be readily generalized for GPRs containing more than two genes related with OR.

(*iv*) More than two genes with a combination of AND and OR relationships are required to enable a reaction *j*. These cases can be mathematically enforced through an appropriate combination of equations (3) and (4). As an example, if three genes *k_1_*, *k_2 _*and *k_3 _*are correlated as (*k_1 _*AND *k_2_*) OR (*k_1 _*AND *k_3_*) to code for the enzyme catalyzing a reaction *j*, then the constraints simulating this relationship can be written as following:

(5) $$\left\{ \begin{array}{l} w_{k_{1}}.LB_{j}\leq v_{j}\leq w_{k_{1}}.UB_{j}\\ (w_{k_{2}}+w_{k_{3}}).LB_{j}\leq v_{j}\leq (w_{k_{2}}+w_{k_{3}}).UB_{j} \end{array}\right.$$

The modified GrowMatch formulation to resolve the GNGs (or GSLs) can now be formulated as following:

$$\begin{array}{l} \underset{{\text{w}}_{\text{j}}}{\text{Minimi}}\text{ze}\;v_{biomass}\\ s.t.\\ \;\left[\begin{array}{ll} \underset{{\text{v}}_{\text{j}}}{\text{Maximize}}\;v_{biomass} & \\ s.t. & \\ \;\sum_{j}s_{ij}v_{j}=0 & \forall \;i\in I\\ \;Appropriate\;GRP\;eqns & \forall \;j\in J\\ \;v_{glu\cos e}\leq v_{glu\cos e}^{\text{uptake limit}} & \\ \;v_{oxygen}\leq v_{oxygen}^{\text{uptake limit}} & \\ \;v_{ATPM}=v_{ATPM}^{\text{maintenance}} & \\ \;LB_{j}\leq v_{j}\leq .UB_{j} & \forall \;j\in J \end{array}\right]\;\\ \;\sum_{j}\left(1-w_{k}\right)\leq n\\ \;w_{k}\in \left\{0,1\right\}\;\;\;\;\;\;\;\forall \;k\in K \end{array}$$

where, *s_ij_*, represents the stoichiometric coefficient of the metabolite *i*, in reaction *j*, *v_biomass _*denotes the biomass flux while $v_{glu\cos e}^{\text{uptake limit}}$, $v_{oxygen}^{\text{uptake limit}}$ and $v_{ATPM}^{\text{maintenance}}$ denote the minimum required glucose and oxygen uptake rates and the non-growth associated ATP for maintenance, respectively. The parameter *n*, represents the allowable number of knock-outs. This bilevel optimization problem can be solved similarly to GrowMatch and SL Finder [18,25] through writing the dual of the inner problem.

### Test of the suggested hypotheses

All the model correction strategies provided by GrowMatch serve as hypotheses that need to be tested to confirm their applicability. Different methods were used to test the validity of each type of modification. Similarly to [18], relaxation of the irreversibility constraints on existing reactions in the model can be checked by using three independent methods. In the first step we check the reversibility of that reaction in *i*AF1260 model of *E. coli *[9] or metabolic models of other organisms. We then query other databases such as MetaCyc [64] about the reversibility of the desired reaction to find out if it is reversible in other organisms. Finally, we examine the reversibility of reactions by computing the value of free Gibbs energy change, *ΔG *[65].

The validity of the added transport reactions to the model are examined by searching the literature to find potential clues about the presence of the suggested transport mechanisms or by querying the databases such as MetaCyc for possibility of presence of those mechanisms in other multi-cellular organisms. The hypotheses for adding new reactions from external databases such as KEGG to the model are tested by performing the bi-directional BLAST between the enzymes catalyzing those reactions and the yeast genome. Consistent with [18,20] we assumed a BLAST expectation value cutoff of 10^-13 ^as the basis to define high sequence similarity. Finally, the gene suppressions are validated by analysis of the gene expression data as well as searching the literature for available evidence. All the global modifications for which we did not find any supporting evidence using the methods mentioned above, were not incorporated into the model and were just added to the list of modifications with no corroborating evidence.

## Authors' contributions

CDM conceived and coordinated the study, participated in its design and helped to draft the manuscript. ARZ performed the simulations and data analysis and drafted the manuscript. Both authors read and approved the final manuscript.

## Supplementary Material

Additional file 1 **The list of model inconsistencies**. The complete list of different types of model inconsistencies for single and double gene perturbations along with literature citations. Click here for file

Additional file 2 **The list of corrections to the model**. The complete list of suggested corrections for mismatches associated with single and double gene perturbations along with supporting evidence. This file also contains details of BLAST analysis for different cases as well as other computations. Click here for file

Additional file 3 **The revised yeast model in SBML format**. Click here for file
